# Effect of *Atractylodes macrocephala* on Hypertonic Stress-Induced Water Channel Protein Expression in Renal Collecting Duct Cells

**DOI:** 10.1155/2012/650809

**Published:** 2012-11-14

**Authors:** Yong Pyo Lee, Yun Jung Lee, So Min Lee, Jung Joo Yoon, Hye Yoom Kim, Dae Gill Kang, Ho Sub Lee

**Affiliations:** ^1^Department of Oriental Medicine and Professional Graduate School of Oriental Medicine, Wonkwang University, Iksan 570-749, Republic of Korea; ^2^Hanbang Body-Fluid Research Center, Wonkwang University, Iksan 570-749, Republic of Korea; ^3^Professional Graduate School of Oriental Medicine, Wonkwang University, Iksan 570-749, Republic of Korea

## Abstract

Edema is a symptom that results from the abnormal accumulation of fluid in the body. The cause of edema is related to the level of aquaporin (AQP)2 protein expression, which regulates the reabsorption of water in the kidney. Edema is caused by overexpression of the AQP2 protein when the concentration of Na^+^ in the blood increases. The rhizome of *Atractylodes macrocephala* has been used in traditional oriental medicine as a diuretic drug; however, the mechanism responsible for the diuretic effect of the aqueous extract from *A. macrocephala* rhizomes (AAMs) has not yet been identified. We examined the effect of the AAM on the regulation of water channels in the mouse inner medullary collecting duct (mIMCD)-3 cells under hypertonic stress. Pretreatment of AAM attenuates a hypertonicity-induced increase in AQP2 expression as well as the trafficking of AQP2 to the apical plasma membrane. Tonicity-responsive enhancer binding protein (TonEBP) is a transcription factor known to play a central role in cellular homeostasis by regulating the expression of some proteins, including AQP2. Western immunoblot analysis demonstrated that the protein and mRNA expression levels of TonEBP also decrease after AAM treatment. These results suggest that the AAM has a diuretic effect by suppressing water reabsorption via the downregulation of the TonEBP-AQP2 signaling pathway.

## 1. Introduction

The kidney tightly regulates the amount of water to be excreted in the urine by reabsorbing up to 99% of the water that is filtered in the glomerulus. Body fluid osmolality is achieved and finely regulated by a number of cellular and molecular processes, including tubular reabsorption of water and sodium through renal water channels and sodium transporters under the tight control of hormones and nerves along with intracellular signaling pathways [1, 2]. Aquaporins (AQPs) are water-selective membrane proteins that are active in tissues that are involved in a high level of water transport in the kidney [3]. At least 8 AQPs are expressed in the kidney and are important with respect to its physiological and pathophysiological aspects [4]. AQPs maintain the extracellular fluid compartment of living cells by regulating water and ion homeostasis [5]. Water transport across kidney tubules and microvessels is important for the reabsorption of water filtered by the glomerulus and for the formation of concentrated urine, which involves countercurrent multiplication and exchange mechanisms and vasopressin-regulated water permeability in the collecting duct. AQP1 is expressed in the cell plasma membrane in the proximal tubule, the thin descending limb of Henle epithelia, and descending vasarecta endothelia [6]. AQP1 facilitates the reabsorption of water in these tubule sections and plays an important role in the countercurrent multiplication process needed to concentrate urine [7, 8]. AQP2 is expressed in the principal cells of the kidney collecting duct, where it is stored in an intracellular compartment located beneath the apical cell membrane. The intracellular trafficking of AQP2 plays an important role in the regulation of urine concentration [9]. AQP3 is constitutively localized in the basolateral membrane of the principal cells of the collecting ducts. This water channel, in parallel with AQP4, facilitates water entry into the interstitium. Vasopressin and aldosterone increase AQP3 expression, whereas insulin decreases AQP3 transcription [10]. In the collecting duct principal cells, its main site of action in the kidney, water reabsorption is regulated by cAMP-dependent translocation of AQP2 from intracellular vesicles primarily into the apical cell membranes [11]. Thus, the expression and targeting of AQP2 are regulated by hypertonic stress in these cells [12, 13]. Transcription factors are also involved in the regulation of AQP2 water permeability. Tonicity-responsive enhancer binding protein (TonEBP) is an essential regulator of AQP2 expression in the principal cells of the renal collecting duct. During antidiuresis, renal medullary cells adapt to the hyperosmotic interstitial environment by increased expression of osmoprotective genes, which is driven by a common transcriptional activator, tonicity-responsive enhancer binding protein (TonEBP) [14]. However, it is not clear that the complicated mechanisms are induced by hypertonic stress in renal medullary cells. 

In a screening study of renal homeostasis involving traditional oriental medicine (TOM), we found that *Atractylodes macrocephala* exhibited significant AQP activity. The *A. macrocephala* Koidz rhizome (Baizhu) is one of the most popular Oriental medicinal plants, with a long history of the treatment of splenic asthenia, anorexia, edema, excessive perspiration, and abnormal fetal movement. Chemical analysis of the *A. macrocephala *rhizomes demonstrated that the main active constituents are sesquiterpenes and acetylenic compounds [15, 16], which have been proven to possess antitumor and anti-inflammatory activities [17, 18]. However, the mechanism responsible for the diuretic effect of *A. macrocephala* rhizomes has not yet been identified.

Our study was performed to determine the possible effects of the aqueous extract from *A. macrocephala* Koidz (AAM) on the water channel regulation response to hyperosmotic stress in mouse inner medullary collecting duct (mIMCD)-3 cells.

## 2. Materials and Methods

### 2.1. Preparation of *A. macrocephala* Extract

Dried *A. macrocephala* rhizomes were purchased from the herbal medicine cooperative association of Chonbuk Province, Korea, in March 2010. A voucher specimen (no. HBF131-05) was deposited at the Hanbang Body-Fluid Research Center, Wonkwang University, Korea. Dried *A. macrocephala* rhizomes (200 g) were boiled in 1.5 L of distilled water for 2 h. The aqueous extract was centrifuged at 890 ×g for 20 min at 4°C and concentrated using a rotary evaporator. The supernatant extract was lyophilized, and a powder was obtained (yield, 21.11 g), which was stored at 4°C until use.

### 2.2. Materials

AQP1, AQP2, AQP3, Na^+^/K^+^-ATPase *α*1-subunit, TonEBP, SMIT, and *β*-actin antibodies were purchased from Santa Cruz Biotechnology (Santa Cruz, CA, USA). Horseradish peroxidase (HRP-) conjugated secondary antibodies were obtained from Stressgen Biotechnologies Corp and Enzo Life Sciences (Farmingdale, NY, USA). 3-Isobutyl-1-methyl-xanthine (IBMX) was purchased from Sigma Chemical Co. (St. Louis, MO, USA). The other reagents used in this study were of the highest purity commercially available.

### 2.3. Cell Line and Culture Conditions

Mouse inner medullary collecting duct (mIMCD-3) cells were obtained from the American Type Culture Collection (ATCC, Manassas, VA, USA). mIMCD-3 cells were routinely cultured in Dulbecco's modified Eagle's/F-12 (DMEM/F-12) medium supplemented with 10% FBS and antibiotics (Gibco & Invitrogen, Carlsbad, CA, USA), in a humidified chamber containing 5% CO_2_ at 37°C. mIMCD-3 cells were analyzed between passages 9 and 11 and were maintained in mIMCD-3 medium. Cells were grown to confluence and cultured without FBS prior to stimulation.

### 2.4. Western Blot Analysis

Cell homogenates (40 *μ*g total protein) were separated by 10% sodium dodecyl sulfate-polyacrylamide gel electrophoresis (SDS-PAGE) and transferred to a nitrocellulose membrane. Blots were washed with H_2_O, blocked with 5% nonfat milk powder in TBST (10 mM Tris-HCl [pH 7.6], 150 mM NaCl, 0.05% Tween-20) for 1 h and incubated with the appropriate primary antibody at the dilutions recommended by the supplier. The membrane was washed, and primary antibodies were detected with an HRP-conjugated secondary antibody. Bands were visualized with enhanced chemiluminescence (Amersham Biosciences, Buckinghamshire, UK). Protein expression levels were determined by analyzing the signals captured on the nitrocellulose membranes by using the Chemi-doc image analyzer (Bio-Rad, Hercules, CA, USA).

### 2.5. Membrane Protein Extraction

A cellular membrane fraction was prepared with Mem-PER (Pierce, Rockford, IL, USA), according to the manufacturer's instructions and as described by Fukuchi et al. [19]. Cells were harvested and incubated with reagent A for 10 min at room temperature with vortexing. Diluted reagent B and reagent C were sequentially added to lysed cells. The samples were incubated at 4°C for 30 min and centrifuged at 10,000 ×g for 3 min. The supernatant was incubated for 10 min at 37°C to separate the membrane protein fraction and then centrifuged at 10,000 ×g for 2 min. The bottom layer was used as the membrane extract.

### 2.6. Preparation of Cytoplasmic and Nucleus Extracts

The cells were rapidly harvested by sedimentation and nuclear and cytoplasmic extracts were prepared on ice as previously described by the method of Mackman et al. [20]. Cells were harvested and washed with 1 mL buffer A (10 mM HEPES, pH 7.9, 1.5 mM MgCl_2_, 19 mM KCl) for 5 min at 600 g. The cells were then resuspended in buffer A and 0.1% NP 40, left for 10 min on ice to lyse the cells and then centrifuged at 600 g for 3 min. The supernatant was saved as cytosolic extract. The nuclear pellet was then washed in 1 mL buffer A at 4,200 g for 3 min, resuspended in 30 *μ*L buffer C (20 mM HEPES, pH 7.9, 25% glycerol, 0.42 M NaCl, 1.5 mM MgCl_2_, 0.2 mM EDTA), rotated for 30 min at 4°C, then centrifuged at 14,300 g for 20 min. The supernatant was used as nucleus extract. The nucleus and cytosolic extracts were then analyzed for protein content using Bradford assay. 

### 2.7. Immunofluorescence

mIMCD-3 cells were seeded on sterile slide coverslips in a 60 mm culture dish and treated with 175 mM NaCl, with or without AAM (1–10 *μ*g/mL) in the culture medium for 1 h. After incubation, the medium was removed; subsequently, the cells were fixed with 4% PFA in PBS at room temperature for 5 min and permeabilized with 0.1% Triton X-100 in PBS for 15 min. The cells were overlaid with 1% BSA in PBS and incubated with the AQP2 antibody (1 : 100, Santa Cruz, CA, USA) incubated at 4°C overnight. The cells were incubated with rhodamine red-conjugated goat anti-rabbit IgG secondary antibody (Invitrogen, CA, USA). The slides were incubated at room temperature for 1 h. Nuclei were stained with 4′,6-diamino-2-phenylindole (DAPI; Molecular Probes, Inc., Eugene, OR, USA), and the apical plasma membrane was labeled by incubating cells with wheat germ agglutinin (WGA; Alexa Fluor 488 conjugate) (Invitrogen, Eugene, OR, USA) diluted 1 : 500 in PBS. Coverslips were mounted with ProLong Gold Antifade Reagents (Molecular Probes, Eugene, OR, USA) onto glass slides and examined under a fluorescence microscope (Axiovision 4, Zeiss, Germany).

### 2.8. Reverse Transcription-Polymerase Chain Reaction (RT-PCR)

Total RNA was isolated from cultured mIMCD-3 cells using a commercially available kit. The yield and purity of the RNA were confirmed by measuring the ratio of the absorbances at 260 and 280 nm. cDNA was prepared from 500 ng RNA by reverse transcription in a final volume of 20 mL in an Opticon MJ Research instrument. The samples were incubated at 37°C for 60 min and at 94°C for 5 min. The following set of primers were used for PCR amplification: AQP1 (forward: 5′-CGGGCTGTCATGTACATCATCGCCCA-3′, reverse: 5′-CCCAATGAACGGCCCCACCCAGAAA-3′), AQP2 (forward: 5′-CACATCAACCCTGCTGTGAC-3′, reverse: 5′-CAGCTGCATGGTCAGGAAGAG-3′), AQP3 (forward: 5′-ACTCCAGTGTGGAGGTGGAC-3′, reverse: 5′-GCCCCTAGTTGAGGATCACA-3′), TonEBP (forward: 5′-AAGACTGAAGATGTTACTCCAATGGAAG-3′, reverse: 5′-AACGTTTGTGCTTGTTCTTGTAGTGG-3′), SMIT (forward: 5′-AGGGAGGCGTTCACCTCAGG-3′, reverse 5′-AACTCCATCACCAGGCGTGGG-3′), and GAPDH (forward: 5′-CAAGGCTGAGAATGGGAAGC-3′, reverse: 5′-AGCATGTGGGAACTCAGATC-3′). Template cDNA and 50 nM primers were added to the PCR Pre-mix according to the manufacturer's instructions (Intron, Korea). The amplification profile was as follows: initial cycling at 94°C for 15 min, followed by 45 cycles of 94°C for 20 s, 60°C for 20 s, and 72°C for 30 s, and a final extension of 72°C for 5 min. The PCR products were resolved by 1.2% agarose gel electrophoresis.

### 2.9. Radioimmunoassay (RIA) for cAMP Measurement

The cAMP levels were determined by a radioimmunoassay, using the protocol developed by Kim et al. [21]. mIMCD-3 cells were incubated for 6 h with AAM in DMEM/F12 medium containing 0.1 mM IBMX and for 10 min in medium supplemented with 175 mM NaCl. After incubation, cAMP production was measured using a *ν*-counter (1480 Automatic Gamma Counter, PerkinElmer, Finland).

### 2.10. Statistical Analysis

All the experiments were repeated at least 3 times. The data were analyzed using one-way ANOVA followed by a Dunnett's test or Student's *t*-test to determine any significant differences. The results were expressed as mean ± S.E. values. *P* < 0.05 was considered as statistically significant.

## 3. Results

### 3.1. Effect of the AAM on Hypertonic Stress-Induced AQPs and Na^+^/K^+^-ATPase Expression in mIMCD-3 Cells

**Figure 1:**
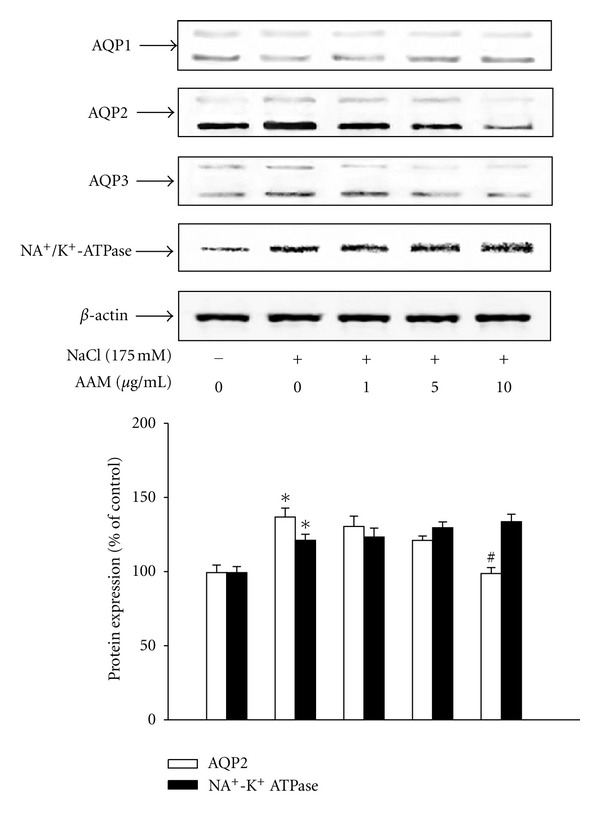
Effect of the AAM on hypertonic stress-induced AQP1, AQP2, AQP3, and Na^+^/K^+^-ATPase expression. (a) mIMCD-3 cells were pretreated with AAM (1–10 *μ*g/mL) for 30 min, followed by stimulation with hypertonic stress (175 mM NaCl) for 1 h. After treatment, the protein was extracted from the cells. AQP1, AQP2, AQP3, and Na^+^/K^+^-ATPase protein levels were determined by western blot analysis. (b) Densitometric analysis of AQP2 and Na^+^/K^+^-ATPase. The values represent the mean ± S.E. values of 3 individual experiments. *P < 0.05 versus control; ^#^ *P* < 0.005 versus NaCl.

Pretreatment with the AAM significantly decreased the hypertonic stress-induced protein expression of AQP1, AQP2, and AQP3. Specifically, pretreatment with the AAM (10 *μ*g/mL) decreased the hypertonic stress-induced increase in AQP2 and AQP3 expression; however, AQP1 expression was not significantly changed by hypertonic stress. In addition, hypertonic stress-induced increase in the Na^+^/K^+^-ATPase *α*1 subunit was not decreased by the AAM (Figure 1). The cytotoxicity of the AAM in mIMCD-3 cells was examined using an MTT assay. mIMCD-3 cells were preincubated with the AAM (1–10 *μ*g/mL) for 24 h, and the MTT assay was performed. The AAM did not alter cell viability at this concentration range (>80% cell viability). The use of the AAM at a concentration greater than 200 *μ*g/mL tended to decrease cell viability, but this change was not significant (data not shown). Therefore, the physiological role of the AAM at noncytotoxic concentrations (less than 200 *μ*g/mL) was examined in mIMCD-3 cells.

### 3.2. Effect of the AAM on Hypertonic Stress-Induced mRNA Expression of AQPs

**Figure 2:**
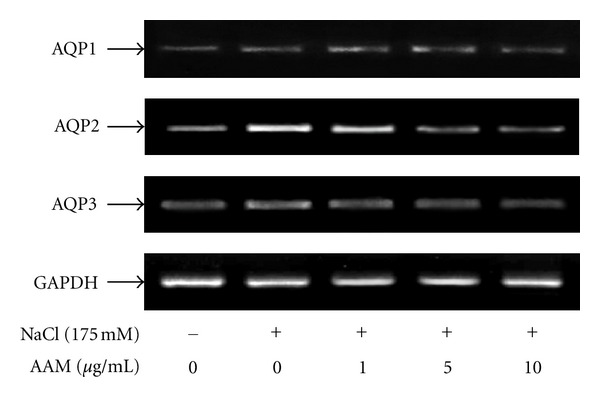
Effect of the AAM on the hypertonic stress-induced increase in AQP mRNA expression. The cells were pretreated with the AAM (1–10 *μ*g/mL) for 30 min and then cultured in medium supplemented with 175 mM NaCl for 1 h. After treatment, total mRNA was extracted from the cells, and AQP1, AQP2, and AQP3 mRNA levels were determined by RT-PCR.

AQP1, AQP2, and AQP3 mRNA expression was measured by RT-PCR. As shown in Figure 2, pretreatment with AAM (1–10 *μ*g/mL) markedly decreased hypertonic stress-induced AQP2 and AQP3 mRNA expression levels, but AQP1 expression did not differ between the NaCl and AAM groups. Therefore, the changes in the mRNA level corresponded to the changes in the protein level.

### 3.3. Effect of the AAM on Hypertonic Stress-Induced Increase in AQP2 Trafficking

**Figure 3:**
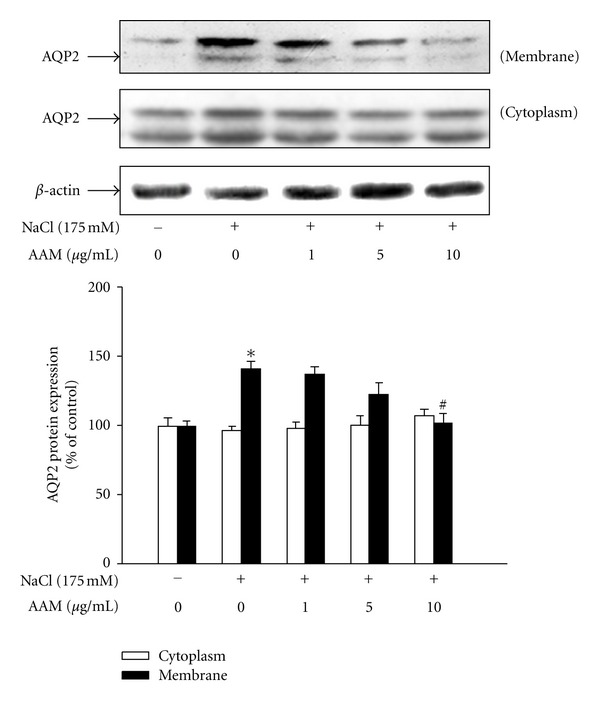
Effect of the AAM on hypertonic stress-induced increase in AQP2 trafficking. mIMCD-3 cells were pretreated with the AAM (1–10 *μ*g/mL) for 30 min and then cultured in medium supplemented with 175 mM NaCl for 1 h. The membrane fractions were extracted, and protein levels were determined by western blot analysis. The values represent the mean ± S.E. values of 3 individual experiments. *P < 0.05, **P < 0.01 versus control; ^#^ *P* < 0.05, ^#^ *P* < 0.05 versus NaCl.

**Figure 4:**
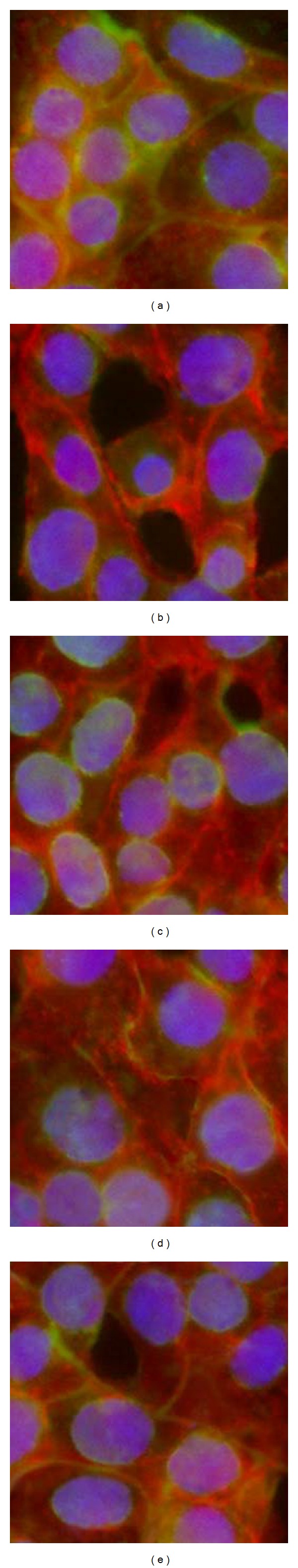
Influence of the AAM on hypertonic stress-induced AQP2 redistribution. WGA (green) was used to stain the plasma membrane, DAPI (blue) was used for the nuclei, and rhodamine (red) was used to counterstain AQP2. (a) Control; (b) hypertonic conditions; (c) cotreatment with 1 *μ*g/mL AAM; (d) cotreatment with 5 *μ*g/mL AAM; (e) cotreatment with 10 *μ*g/mL AAM.

 Arginine vasopressin (AVP-) induced water osmosis is dependent on the insertion of the AQP2-containing vesicle into the apical membrane [3]. To determine whether the AAM decreases the membrane-targeted insertion of AQP2, the apical membrane expression of AQP2 was examined. The addition of medium supplemented with 175 mM NaCl strongly enhanced the apical membrane insertion of AQP2, whereas AAM decreased insertion by 30% (Figure 3). To determine whether the translocation observed in mIMCD-3 cells also reflects a membrane insertion of AQP2, surface membrane proteins were biotinylated before and after stimulation with NaCl. Biotinylated proteins, which were restricted to plasma membrane proteins, were captured using streptavidin-conjugated beads. NaCl produced a significant increase in the biotinylation of AQP2, consistent with an increase in the membrane density of Figure 3 (see Figure 1 in the Supplementary Materials available online at doi:10.1155/2012/650809). AAM markedly attenuated NaCl-induced AQP2 biotinylation. Thus, this result confirms that hypertonic stress-induced translocation of AQP2 in mIMCD-3 cells reflects apical membrane insertion. For immunofluorescence microscopy, the cells were fixed and incubated with Alexa Fluor 488-tagged WGA to mark the apical surface; subsequently, they were permeabilized and labeled with AQP2. Microscopy showed that AQP2 (red) was predominantly intracellular in control and isotonic cells and localized to the apical membrane with the plasma membrane marker after stimulation with 175 mM NaCl. However, pretreatment with AAM decreased NaCl-induced AQP2 plasma membrane expression in mIMCD-3 cells (Figure 4). 

### 3.4. Effect of the AAM on Hypertonic Stress-Related Signal Pathways

**Figure 5:**
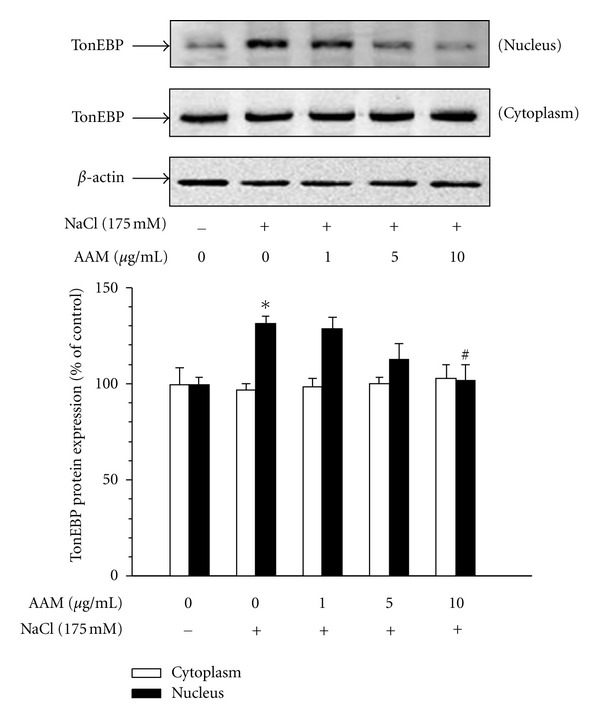
Effect of the AAM on hypertonic stress-induced TonEBP expression and translocation into the nucleus in mIMCD-3 cells. mIMCD-3 cells were pretreated with the AAM (1–10 *μ*g/mL) for 30 min. Cytoplasm and the nuclear fractions were extracted, and the protein levels were determined by western blot analysis. The bands indicate TonEBP (165 kDa). The blots are representative of 3 independent experiments (a) and densitometric quantification (b) of TonEBP. The values represent the mean ± S.E. values of 3 individual experiments. *P < 0.05 versus control; ^#^ *P* < 0.05 versus NaCl.

**Figure 6:**
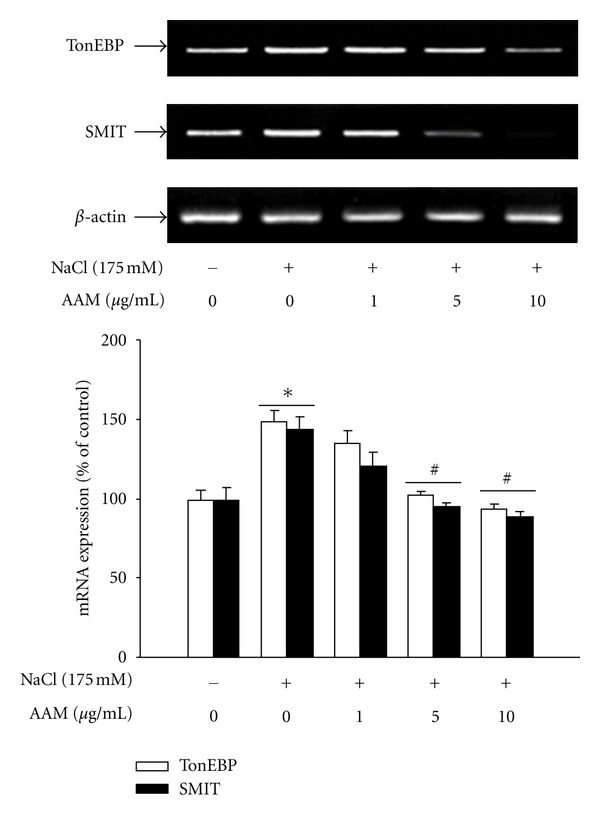
Effect of the AAM on hypertonic stress-induced TonEBP, and SMIT mRNA expression. The cells were pretreated with AAM (1–10 *μ*g/mL) for 30 min and then cultured in medium supplemented with 175 mM NaCl for 1 h. TonEBP, and SMIT mRNA levels were determined by RT-PCR. The values represent the mean ± S.E. values of 3 individual experiments. *P < 0.05 versus control; ^#^ *P* < 0.05 versus NaCl.

 To measure changes in the activity of the TonEBP protein, we measured protein expression in mIMCD-3 cells (Figure 5). Confluent mIMCD-3 cells were pretreated with AAM and then treated with 175 mM NaCl. The hypertonic solution significantly increased the TonEBP protein level. The TonEBP in the nuclear fractions of mIMCD-3 decreased after treatment with AAM in a dose-dependent manner. To measure changes in the activity of SMIT and TonEBP, we measured their mRNA expression in mIMCD-3 cells (Figure 6). Confluent mIMCD-3 cells were pretreated with the AAM and then treated with 175 mM NaCl. The hypertonic solution significantly increased mRNA expression, whereas treatment with the AAM decreased the expression of SMIT and TonEBP. 

### 3.5. Involvement of cAMP/PKA Pathway in the Inhibitory Effect of the AAM on Hypertonic Stress-Induced AQP2 Expression

**Figure 7:**
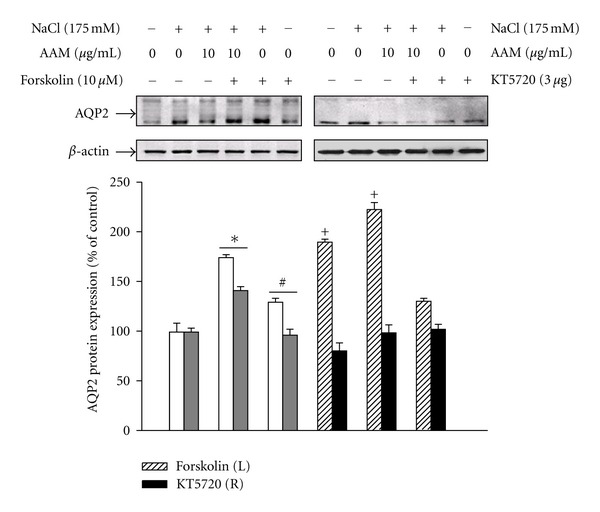
Effects of the AAM with forskolin (adenylate cyclase activator) or KT5720 (PKA inhibitor) on hypertonic stress-induced AQP2 expression. The cells were pretreated with forskolin (10 *μ*M), or KT5720 (3 *μ*M) with/without AAM (10 *μ*g/mL), and then cultured in medium supplemented with 175 mM NaCl for 1 h. After treatment, the protein was extracted from the cells, and AQP2 expression was determined by western blot analysis. The values represent the mean ± S.E. values of 3 individual experiments. *P < 0.05 versus control; ^#^ *P* < 0.005 versus NaCl alone; ^+^ *P* < 0.05 versus the AAM with NaCl.

To evaluate the involvement of cAMP/protein kinase A (PKA) pathway on the inhibitory effects of the AAM during hypertonic stress-induced AQP2 expression, the cells were pretreated with forskolin, an adenylate cyclase activator, or AAM and then with 175 mM NaCl. As shown in Figure 7, pretreatment with forskolin significantly enhanced AQP2 expression under hypertonic stress. In contrast, pretreatment with AAM showed an effect similar to that of KT5720, a cell-permeable-specific competitive inhibitor of PKA, which decreased hypertonic stress-induced AQP2 expression. 

**Figure 8:**
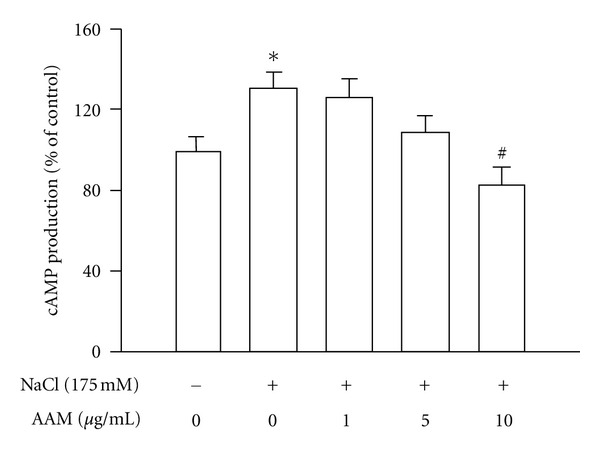
Effects of the AAM on hypertonic stress-induced suppression of cAMP in mIMCD-3. The cells were pretreated with the AAM (1–10 *μ*g/mL) for 30 min in DMEM/F12 medium containing 0.1 M IBMX and then cultured in medium supplemented with 175 mM NaCl for 1 h. The values represent the mean ± S.E. values of 3 individual experiments. *P < 0.05 versus control; ^#^ *P* < 0.05 versus NaCl.

To examine the role of cAMP in AQP2 trafficking, mIMCD-3 cells were pretreated with the AAM and then treated with NaCl. NaCl significantly increased cAMP content after 1 min, and the hypertonic stress-induced cAMP production was markedly inhibited by AAM (Figure 8). 

## 4. Discussion

TOM, recognized as one of the numerous complementary and alternative medicine modalities in the West, is very popular in the general population of the Eastern countries. Several special herbal products with low levels of side effects are of great interest as therapy for renal failure [21–24]. This study demonstrates the beneficial effect of the AAM in the treatment of water imbalance during *in vitro* hypertonic stress. The classical cAMP/PKA pathway and the recently discovered TonEBP are involved in hypertonic stress-induced AQP2 expression. The AAM may block these signal pathways, resulting in renal homeostasis regulation. Water is driven by an osmotic gradient and moves apically into the cell via AQP2; subsequently, it exits across the basolateral membrane via AQP3 and/or AQP4 [25]. The clinical importance of AQP2 is illustrated by imbalances in body fluid homeostasis that arise from dysregulated AQP2 expression. Decreased AQP2 expression, manifested in nephrogenic diabetes insipidus (NDI), leads to an inability to maximally concentrate urine. NDI patients consequently excrete large amounts of hypotonic urine (up to 20 liters per day) that must be compensated by excessive fluid uptake [26]. Conversely, AQP2 overexpression associated with congestive heart failure, and pregnancy, leads to water retention and increased extracellular fluid volume [27]. Thus, downregulation of the expression of this water channel in the presence of excess salt could contribute to the increased urine flow rate. In this study, hypertonic stress (650 mosmol/kg NaCl) induces an increase in AQP2 and AQP3 expressions; however, their expressions were attenuated by pretreatment with the AAM. The renal collecting duct is involved in urine concentration via a process that is regulated by the antidiuretic hormone [28]. Vasopressin is known to upregulate both AQP2 and AQP3 in the collecting duct, and there is clear evidence that AQP2 and AQP3 are related to changes in vasopressin and water balance [4, 29]. Thus, this result suggests that AAM regulates vasopressin and water balance under hypertonic stress conditions. Upon activation of PKA, AQP2 is phosphorylated and is rapidly redistributed from intracellular vesicles to the apical membrane of the collecting duct principal cells [30]. Na^+^/K^+^-ATPase *α*1-subunit also has a specific effect on the hypertonic condition; however, pretreatment with AAM does not alter Na^+^/K^+^-ATPase *α*1-subunit expression. Therefore, we suggest that the AAM specifically regulates hypertonic stress-induced AQP2 expression in the apical membrane. This proposal is supported by immunofluorescence results, which demonstrated that rhodamine-conjugated AQP2 is predominantly localized in the apical membrane after NaCl stimulation. However, pretreatment with AAM decreased NaCl-induced AQP2 plasma membrane insertion in IMCD-3 cells. In addition, AAM significantly decreased hypertonicity-induced AQP2 expression in membrane protein and biotinylated proteins. 

We demonstrated that AAM exhibits a primary regulatory role in renal water excretion under hypertonic stress. However, a recent study reported that increased urine output of excess glucocorticoid is not related to alterations in renal AQP water channels [31]. There may be several potential mechanisms underlying the regulation of AQP expression by the AAM under hypertonic stress in mIMCD-3 cells. In this study, pretreatment of AAM decreased hypertonic stress-induced TonEBP nuclear and mRNA expression. Mice expressing dominant-negative restriction of TonEBP to the kidney collecting duct show decreased levels of both UT-A1 urea transporter and AQP2 mRNA [32]. The extent of decreased AQP2 expression is similar in the presence or absence of vasopressin, indicating that TonEBP acts independently of vasopressin-mediated events. In the surviving mice harboring a functionally inactive TonEBP gene, the kidney protein expression levels of TonEBP-targeted genes such as aldose reductase, sodium-myo-inositol cotransporter, and taurine transporter together with AQP2 are lower than those in the wild-type kidney [33]. These results suggest that AAM improves dehydration-induced water imbalance via inhibition of the TonEBP signal pathways in the inner medullary collecting ducts. Currently, the condition underlying hypertonicity remains undefined even though a similar natriuresis is seen following infusion of hypertonic saline. Further studies will be required to develop a precise experimental *in vivo* model for natriuresis or diuresis. 

It has been suggested that hypertonicity depends on the “classical” cAMP/PKA pathway. In the collecting duct, water permeability is chiefly controlled by AVP, leading to Gs*α*/adenylyl cyclase activation, increased intracellular cAMP concentration, and cAMP/PKA activation. This event induces rapid AQP2 translocation from intracellular storage vesicles to the apical membrane responsible for enhanced apical water permeability [3, 29]. In our study, AAM treatment blocked hypertonic stress-induced cAMP content in a dose-dependent manner. We had previously reported that AQP2 is regulated by the cAMP in osmotic stress response pathway [34]. However, this finding is inconsistent with previous studies by Hasler [26] in which hypertonicity does not increase cAMP concentration or cAMP response element-binding protein (CREB) phosphorylation. PKA is necessary for increase in TonEBP/OREBP-mediated transcriptional activity in response to hypertonicity, and hypertonicity-induced activation of PKA is cAMP independent in HepG2 cells [35]. In contrast, PKA-independent cAMP regulation of AQP2 expression has been suggested [13]. We postulate that the modulatory effects of cAMP/PKA-mediated AQP2 expression by hypertonicity are dependent on various incubation time or cell type. Further study of AQP2-related mechanism should prove to be useful for elucidating the complicated steps in the cAMP/PKA pathway under hypertonicity. AAM exhibited a similar effect on the PKA inhibitor, which decreased hypertonic stress-induced AQP2 expression. These results suggested that AAM decreased apical AQP2 expression throughout the inhibition of cAMP/PKA signal pathway and direct/indirect involvement of TonEBP under hypertonic stress in IMCD-3 cells. 

Cirrhosis induced by carbon tetrachloride may be associated with the late decompensated stage of liver cirrhosis, characterized by sodium retention, edema, and ascites [34, 36]. Thus, the downregulation of AQP2 observed in milder forms of cirrhosis may represent a compensatory mechanism to prevent development of water retention. In contrast, the increased levels of vasopressin seen in severe “noncompensated” cirrhosis with ascites may induce inappropriate upregulation of AQP2 that in turn is involved in the development of water retention. The inhibitory effect of the AAM on the AQP2 water channel in an *in vitro* model of excess salt concentration suggests a possible approach for cirrhosis treatment. These results provide evidence that *A. macrocephala* rhizomes could be used to regulate water balance under various pathophysiological conditions in the kidney. 

## Supplementary Material

Effect of AAM on hypertonic stress-induced AQP2 in plasma membrane insertion.

## References

[1] Klussmann E, Maric K, Rosenthal W (2000). The mechanisms of aquaporin control in the renal collecting duct. *Reviews of Physiology Biochemistry and Pharmacology*.

[2] Saad S, Agapiou DJ, Chen XM, Stevens V, Pollock CA (2009). The role of Sgk-1 in the upregulation of transport proteins by PPAR-*γ* agonists in human proximal tubule cells. *Nephrology Dialysis Transplantation*.

[3] Nielsen S, Chou CL, Marples D, Christensen EI, Kishore BK, Knepper MA (1995). Vasopressin increases water permeability of kidney collecting duct by inducing translocation of aquaporin-CD water channels to plasma membrane. *Proceedings of the National Academy of Sciences of the United States of America*.

[4] Kwon TH, Laursen UH, Marples D (2000). Altered expression of renal AQPs and Na^+^ transporters in rats with Lithium-induced NDI. *American Journal of Physiology—Renal Physiology*.

[5] Hozawa S, Holtzman EJ, Ausiello DA (1996). cAMP motifs regulating transcription in the aquaporin 2 gene. *American Journal of Physiology—Cell Physiology*.

[6] Verkman AS (2008). Dissecting the roles of aquaporins in renal pathophysiology using transgenic mice. *Seminars in Nephrology*.

[7] Pallone TL, Edwards A, Ma T, Silldorff EP, Verkman AS (2000). Requirement of aquaporin-1 for Nacl-driven water transport across descending vasa recta. *Journal of Clinical Investigation*.

[8] King LS, Choi M, Fernandez PC, Cartron JP, Agre P (2001). Defective urinary concentrating ability due to a complete deficiency of aquaporin-1. *New England Journal of Medicine*.

[9] Takata K, Matsuzaki T, Tajika Y, Ablimit A, Hasegawa T (2008). Localization and trafficking of aquaporin 2 in the kidney. *Histochemistry and Cell Biology*.

[10] Kwon TH, Nielsen J, Masilamani S (2002). Regulation of collecting duct AQP3 expression: response to mineralocorticoid. *American Journal of Physiology—Renal Physiology*.

[11] Klussmann E, Rosenthal W (2001). Role and identification of protein kinase A anchoring proteins in vasopressin-mediated aquaporin-2 translocation. *Kidney International*.

[12] Rauchman MI, Nigam SK, Delpire E, Gullans SR (1993). An osmotically tolerant inner medullary collecting duct cell line from an SV40 transgenic mouse. *American Journal of Physiology—Renal Fluid and Electrolyte Physiology*.

[13] Umenishi F, Narikiyo T, Vandewalle A, Schrier RW (2006). cAMP regulates vasopressin-induced AQP2 expression via protein kinase A-independent pathway. *Biochimica et Biophysica Acta*.

[14] Hasler U, Jeon US, Kim JA (2006). Tonicity-responsive enhancer binding protein is an essential regulator of aquaporin-2 expression in renal collecting duct principal cells. *Journal of the American Society of Nephrology*.

[15] Huang BS, Sun JS, Chen ZL (1992). Isolation and identification of atractylenolide Vl from Atractylodes macrocephala Koidz. *Acta Botanica Brasilica*.

[16] Chen ZL (1987). The acetylenes from Atractylodes macrocephala. *Planta Medica*.

[17] Li CQ, He LC, Dong HY, Jin JQ (2007). Screening for the anti-inflammatory activity of fractions and compounds from Atractylodes macrocephala koidz. *Journal of Ethnopharmacology*.

[18] Huang HL, Chen CC, Yeh CY, Huang RL (2005). Reactive oxygen species mediation of Baizhu-induced apoptosis in human leukemia cells. *Journal of Ethnopharmacology*.

[19] Fukuchi J, Hiipakka RA, Kokontis JM (2004). Androgenic suppression of ATP-binding cassette transporter A1 expression in LNCaP human prostate cancer cells. *Cancer Research*.

[20] Mackman N, Brand K, Edgington TS (1991). Lipopolysaccharide-mediated transcriptional activation of the human tissue factor gene in THP-1 monocytic cells requires both activator protein 1 and nuclear factor *κ*B binding sites. *Journal of Experimental Medicine*.

[21] Kim SZ, Kim SH, Park JK, Koh GY, Cho KW (1998). Presence and biological activity of C-type natriuretic peptide-dependent guanylate cyclase-coupled receptor in the penile corpus cavernosum. *Journal of Urology*.

[22] Ahn CB, Song CH, Kim WH, Kim YK (2002). Effects of Juglans sinensis Dode extract and antioxidant on mercury chloride-induced acute renal failure in rabbits. *Journal of Ethnopharmacology*.

[23] Lee HW, Kim DW, Phapale PB (2011). In vitro inhibitory effects of Wen-pi-tang-Hab-Wu-ling-san on human cytochrome P450 isoforms. *Journal of Clinical Pharmacy and Therapeutics*.

[24] Zhu R, Chen YP, Deng YY (2011). Cordyceps cicadae extracts ameliorate renal malfunction in a remnant kidney model. *Journal of Zhejiang University SCIENCE B*.

[25] Van Balkom BWM, Van Raak M, Breton S (2003). Hypertonicity is involved in redirecting the aquaporin-2 water channel into the basolateral, instead of the apical, plasma membrane of renal epithelial cells. *Journal of Biological Chemistry*.

[26] Hasler U (2009). Controlled aquaporin-2 expression in the hypertonic environment. *American Journal of Physiology—Cell Physiology*.

[27] Fujita N, Ishikawa SE, Sasaki S (1995). Role of water channel AQP-CD in water retention in SIADH and cirrhotic rats. *American Journal of Physiology—Renal Fluid and Electrolyte Physiology*.

[28] Combet S, Gouraud S, Gobin R (2008). Aquaporin-2 downregulation in kidney medulla of aging rats is posttranscriptional and is abolished by water deprivation. *American Journal of Physiology—Renal Physiology*.

[29] Nielsen S, Frøkiær J, Marples D, Kwon TH, Agre P, Knepper MA (2002). Aquaporins in the kidney: from molecules to medicine. *Physiological Reviews*.

[30] Breyer JA, Bain RP, Evans JK (1996). Predictors of the progression of renal insufficiency in patients with insulin-dependent diabetes and overt diabetic nephropathy. *Kidney International*.

[31] Li C, Wang W, Summer SN, Falk S, Schrier RW (2008). Downregulation of UT-A1/UT-A3 is associated with urinary concentrating defect in glucocorticoid-excess state. *Journal of the American Society of Nephrology*.

[32] Nakayama Y, Peng T, Sands JM, Bagnasco SM (2000). The TonE/TonEBP pathway mediates tonicity-responsive regulation of UT-A urea transporter expression. *Journal of Biological Chemistry*.

[33] López-Rodríguez C, Antos CL, Shelton JM (2004). Loss of NFAT5 results in renal atrophy and lack of tonicity-responsive gene expression. *Proceedings of the National Academy of Sciences of the United States of America*.

[34] Lee SM, Lee YJ, Yoon JJ, Kang DG, Lee HS (2012). Effect of Poria cocos on hypertonic stress-induced water channel expression and apoptosis in renal collecting duct cells. *Journal of Ethnopharmacology*.

[35] Ferraris JD, Persaud P, Williams CK, Chen Y, Burg MB (2002). cAMP-independent role of PKA in tonicity-induced transactivation of tonicity-responsive enhancer/osmotic response element-binding protein. *Proceedings of the National Academy of Sciences of the United States of America*.

[36] Ginés P, Berl T, Bernardi M (1998). Hyponatremia in cirrhosis: from pathogenesis to treatment. *Hepatology*.

