# Supplementary material for: Effect of Atractylodes macrocephala on Hypertonic Stress-Induced Water Channel Protein Expression in Renal Collecting Duct Cells
Source: Evid Based Complement Alternat Med. 2012 Nov 14;2012:650809. doi: 10.1155/2012/650809 (PMC3522508; doi:10.1155/2012/650809)
Supplement: Supplementary file 1 — Effect of AAM on hypertonic stress-induced AQP2 in plasma membrane insertion. [file 650809.f1.docx]

**Effect of AAM on hypertonic stress-induced AQP2 in plasma membrane insertion**

**Biotinylation of mIMCD-3 surface membrane protein.**

mIMCD-3 cell were cultured to confluence on polylysine-coated 100-mm dishes and treated with 175 mM NaCl, with or without AAM (10 μg/ml) in the culture medium for 30 min and vasopressin (AVP, 10 ng/ml) for 1 h, respectively. Cells were harvested with ice-cold PBS and centrifugation at 50 g for 10s at 4°C, the supernatant was discarded and the pellet was washed three times at 4°C with ice-cold biotinylation buffer solution (in mM: 215 NaCl, 4 KCl, 2.5 Na_2_HPO_4_, 2 CaCl_2_, 1.2 MgSO_4_, 5.5 glucose, and 10 triethanolamine, pH 7.4) without biotin. Final pellet was resuspended in 1 ml ice-cold biotinylation solution containing 3.0 mg/ml of Sulfo-NHS-SS-Biotin (Product No. 21331, Pierce) and incubated for 60 min at 4°C with gentle horizontal motion to ensure mixing. The suspensions were then washed three times with ice-cold biotin quenching solution (0.1 mM CaCl_2_, 1 mM MgCl_2_, and 260 mM glycine in PBS, pH 7.4) followed by gentle mixing for 20 min at 4°C with the same solution. After washing with ice-cold lysis buffer (150 mM NaCl, 5 mM EDTA, and 50 mM Tris, pH 7.4) containing protease inhibitors (1 μg/ml of leupeptin and 0.1 mg/ml of phenylmethylsulfonyl fluoride) without detergent three times, the suspension was solubilized in 1 ml of lysis buffer (which included 1% NP-40) for 60 min at 4°C with gentle motion. The samples were centrifuged at 14,000g for 10 min at 4°C, and 900 μl of the supernatant was added to 200 μl of streptavidin beads (ImmunoPure, No. 20349, Pierce). The bead suspension was incubated overnight (16 h) at 4°C with continuous agitation. After washing the beads three times with lysis buffer containing 1% NP-40, twice with high-salt wash buffer (500 mM NaCl, 5 mM EDTA, and 50 mM Tris, pH 7.5), and once with no-salt wash buffer (10 mM Tris, pH 7.5) at 4°C, biotinylated proteins were eluted from the beads with 100 μl of 1.5% SDS in Laemmli sample buffer incubated at 60°C for 15 min before separation by SDS-PAGE.

**Results**

Arginine vasopressin (AVP) and 175 mM NaCl strongly enhanced the plasmalemma accumulation of AQP2, whereas AAM decreased accumulation. This result suggested that AAM blocked hypertonic stress-induced AQP insertion in apical membrane of IMCD-3 cells.

**AQP2**

**29**

**35**

**AVP (50 ng/ml)**

**- + - -**

**NaCl (175 mM)**

**- - + +**

**AAM (10 μg/ml)**

**- + - +**


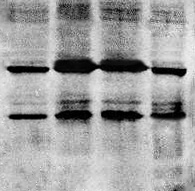


**a**

**b**

**c**

**d**

Supplement 1. Effect of the AAM on hypertonic stress-induced increases AQP2 experssion in plasmalemmal protein of mIMCD-3 cells. Cells were pretreated with the AAM (10 μg/ml) for 30 min and then cultured in medium supplemented with 175 mM NaCl for 1 h. Western blot of biotinylated proteins from mIMCD-3 suspension were probed with anti-AQP2. (a) Control; (b) AVP; (c) hypertonic condition; (d) cotreatment with 10 μg/ml AAM.
